# Interaction of Nanomaterials with Nucleic Acids and Their Applications in Nucleic Acid Analysis

**DOI:** 10.7150/ijbs.113309

**Published:** 2025-06-09

**Authors:** Jiale Wang, Kai Li, Fukai Li, Xinran Li, Jian Zhou, Mengrui Yang, Xiao Zhang, Mengyu Wang, Liang Li

**Affiliations:** 1 School of Life Science and Technology, Changchun University of Science and Technology, Changchun 130022, China.; 2 State Key Laboratory for Quality and Safety of Agro-Products, Institute of Quality Standard and Testing Technology for Agro-products, Chinese Academy of Agricultural Sciences, Beijing 100081, China.; 3 Biotechnology Research Institute, Chinese Academy of Agricultural Sciences, Beijing 100081, China.

**Keywords:** nucleic acid, nanotechnology, extraction, interaction modes

## Abstract

Nucleic acid analysis technology is the key to cracking the genetic information of life, which is very important for insight into disease diagnosis, drug development, food safety and environmental monitoring. The successful implementation of nucleic acid analysis depends on efficient and accurate nucleic acid sample purification technology. Traditional nucleic acid extraction methods are not only time-consuming and difficult to handle but also require skilled operators. Nanotechnology is gradually innovating nucleic acid extraction, simplifying the process and promoting biological science into a new era. The interaction modes between nanomaterials and nucleic acid molecules are diverse, including electrostatic interaction, covalent binding (direct covalent bonding, biotin-avidin system, disulfide bond connection, coordination bond, azide-alkyne click reaction, EDC/NHS coupling), π-π stacking effect, hydrogen bond formation, hydrophobic interaction and ion exchange. Among them, electrostatic interaction and covalent binding are particularly common and widely used. In addition, integrating nanomaterials into advanced monitoring systems such as microfluidic chips and biosensors provides strong support for the innovation of nucleic acid detection technology. The purpose of this paper is to comprehensively explain the basic principles and related molecular mechanisms of the interaction between nucleic acids and nanomaterials and to demonstrate their effectiveness in practical applications through specific examples for each interaction mode. Finally, we will review the latest progress of nanomaterial application in nucleic acid analysis, aiming to provide valuable references and inspirations for future research and development in this field.

## 1. Introduction

As a key technology to cracking the genetic code of life, nucleic acid analysis is of great significance for understanding biological genetic characteristics, diagnosing genetic and infectious diseases, studying biological evolution and diversity, accelerating drug development, food safety and environmental protection. It is an indispensable core tool in modern biology, medicine and other fields. The success of nucleic acid analysis largely depends on the method of purifying nucleic acids from samples 1. The traditional nucleic acid purification methods are not only time-consuming and laborious but also require the professional skills of operators, which undoubtedly limits their application in large-scale experiments and rapid detection 2, 3. The vigorous development of nanotechnology has brought revolutionary changes to nucleic acid purification. As a kind of material with a unique microstructure, the characteristic size of nanoparticles (NPs) is usually defined in the range of several nanometers (usually 1 to 10 nanometers), which endows them with a series of extraordinary physical and chemical properties 4, 5. These properties make nanomaterials (NMs) occupy a pivotal position in the rapidly developing field of nucleic acid analysis, and have demonstrated excellent performance and an indispensable role in many fields of human activity 6, 7.

**Figure 1:**
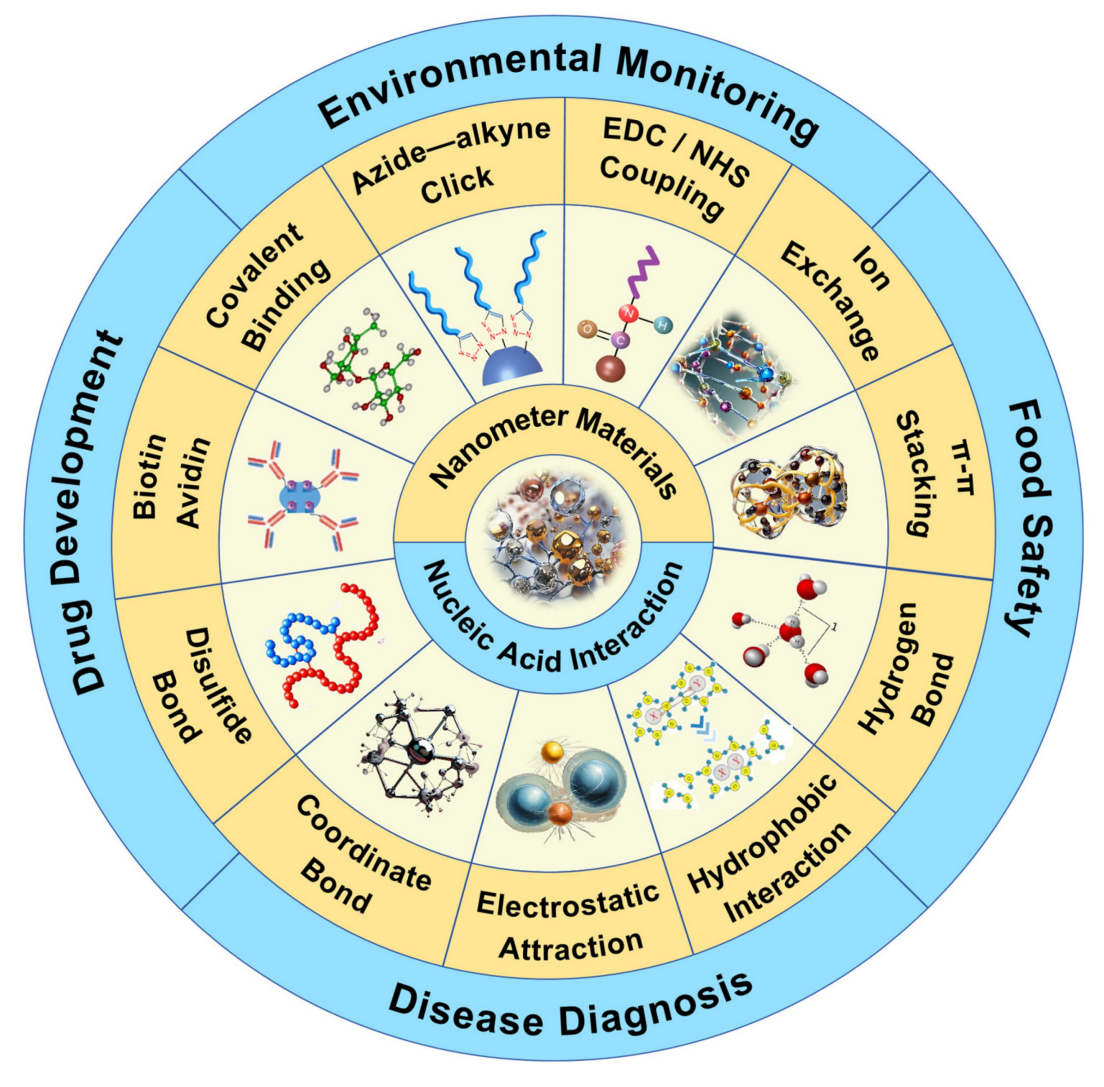
Overview of the interaction of NMs with nucleic acids and their application in nucleic acid analysis. At present, the interactions between nucleic acids and NMs mainly include electrostatic interaction, covalent binding (direct covalent binding, biotin-avidin, disulfide bond, coordination interaction, azide-alkyne click reaction, EDC/NHS coupling), π-π stacking, hydrogen bond formation, hydrophobic interaction, ion exchange, and the main application fields include environmental monitoring and protection, disease diagnosis, food safety detection, drug research and development.

Nucleic acid is composed of nucleotide units, including negatively charged phosphate groups, pentose and bases 8, 9. The negative charge density of the nucleic acid is mainly concentrated on the main chain composed of its phosphate group, which enables the nucleic acid to form hydrogen bonds and electrostatic interactions with the NMs. At the same time, the bases of the nucleic acid can also be adsorbed on the surface of the NMs 10, 11. In addition, complex interactions such as hydrophobic interactions and covalent binding may also occur between nucleic acids and NMs. These interactions together constitute the diversity and complexity of the binding of nucleic acids to NMs **(Figure 1)**. NMs with unique chemical and physical properties can be synthesized, its huge surface area provides a wealth of binding sites for DNA, thereby achieving efficient loading. More importantly, NPs can also effectively protect nucleic acids from nuclease degradation, further improving the stability and safety of nucleic acid extraction 12, 13.

This paper aims to comprehensively review the basic principles and related molecular mechanisms of the interaction between nucleic acids and NMs and enumerate practical application cases for each interaction mode **(Table 1)**. Finally, we will summarize the current application progress of NMs in the field of nucleic acid analysis to provide useful reference and inspiration for future research and development.

## 2. Electrostatic Interaction between Nanomaterials and Nucleic Acids

**Figure 2:**
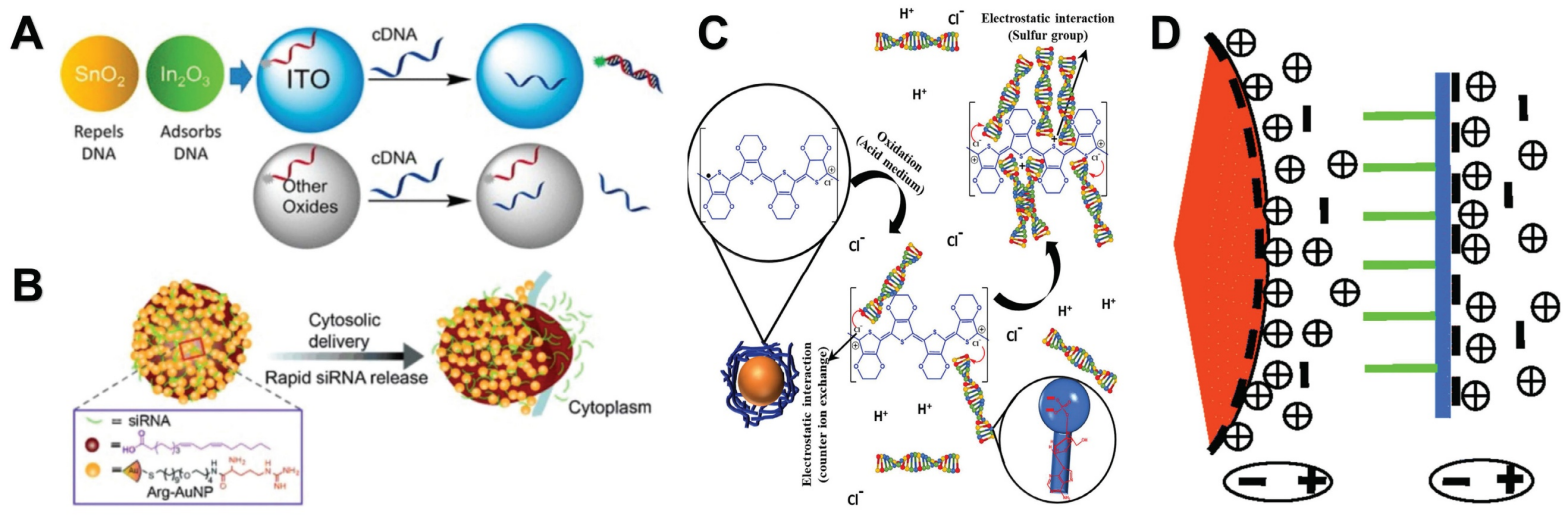
Electrostatic interaction between NMs and nucleic acids. **A**. DNA Adsorption by Indium tin oxide NPs. “Reproduced with permission from American Chemical Society publisher (journal citation 14)”. **B**. siRNA is complexed with cationic arginine-functionalized gold nanoparticles by electrostatic interactions, the complex rapidly delivers siRNA into the cytosol through membrane fusion, “Reproduced with permission from John Wiley and Sons publisher (journal citation 37)”. **C**. Interaction mechanism of PEDOT adsorption of DNA. “Reproduced with permission from Elsevier publisher (journal citation 43)”. **D**. A schematic illustration of the interaction between negatively charged AuNP and single stranded DNA, with the orange wedge representing AuNP, the green line representing the DNA bases, and the blue line representing the phosphate skeleton. “Reproduced with permission from American Chemical Society publisher (journal citation 44)”.

Nucleic acid is a biomolecule composed of negatively charged phosphate groups, pentose and base pairs that can be paired with each other. It can interact with positively charged molecules by electrostatic interaction. Therefore, in the process of using NMs to analyze nucleic acids, NMs with positive surface charges can be used to interact directly with nucleic acids through electrostatic interaction. Here we review several application examples of electrostatic interaction between NPs with positive surface charges and nucleic acids. For example, Indium tin oxide (ITO) NPs can adsorb the phosphate backbone of nucleic acid by electrostatic interaction. At low pH, the surface of ITO is positively charged, which enhances the adsorption of DNA. DNA adsorption is also affected by nucleic acid sequence and length. When the single-stranded nucleic acid adsorbed on the ITO surface becomes double-stranded, DNA will be desorbed, but this phenomenon does not occur on other metal oxides such as CeO_2_, TiO_2_ and Fe_3_O_4_ **(Figure 2A)** 14. Gold nanoparticles (AuNPs) can bind to single-stranded DNA (ssDNA) and RNA, and the degree of binding depends on the charge of the NPs and the sequence and type of the nucleic acid 15. DNA can be adsorbed on the plane of organic clay and montmorillonite by electrostatic force. Cai *et al.* found that it is easier to elute DNA by AuNPs than other NMs 16. Nucleic acid can also produce electrostatic binding interaction with the surface of spinel ferrite. Iqubal *et al.* found that it has the greatest adsorption effect at low pH (4.0) 17. In addition, enamelin 18 and nickel ferrite 19 can also be used as raw materials for NMs, binding to nucleic acid molecules by electrostatic interaction. Electrostatic interaction between NMs and nucleic acids have shown great potential in the field of nucleic acid analysis. By virtue of their unique electrostatic adsorption properties, they can quickly and accurately capture nucleic acids from biological samples with complex components and numerous interference factors. At the same time, these NMs have good compatibility and integration, and can be easily integrated into advanced analytical equipment such as microfluidic chips, significantly improving the efficiency and accuracy of nucleic acid analysis.

However, due to the weak electrostatic interaction and the small amount of charge carried by NMs, the electrostatic interaction between NMs and nucleic acids is often susceptible to interference. Therefore, many researchers use other NMs modified on the surface of NMs to improve the electrostatic interaction between NMs and nucleic acids 20. Silica is one of the most widely used NMs. When there is a certain concentration of cations in the solution, nucleic acids are capable of adhering to the surface of silica-coated magnetic particles, where the negatively charged phosphate backbone of the nucleic acid establishes a cationic bridge with the negatively charged particle surface 21, 22. It has shown that within 30 minutes, silica-coated magnetic particles can effectively extract Hepatitis B virus and Hepatitis C virus nucleic acids from serum 23, as well as total nucleic acids of RNA viruses/viroids from lily or grape leaf samples 24, and even perform simultaneous isolation of DNA and RNA from hepatocellular carcinoma tissues 25. Based on silica modification, some binary modification materials have also been developed. For example, Tiwari *et al.* developed a Fe_3_O_4_@silica@chitosan nanoparticle to successfully isolate genomic DNA from human saliva 26. Ali *et al.* proposed a magnet nanomaterial coated with silica. The silica coating is coupled with a triethylene glycol-spaced glycosyl imidazole, and the imidazolyl group interacts with the nucleic acid 27.

The polyvalent cationic agent polyethyleneimine (PEI) is one of the most widely used modifiers 28. Some researchers have found that the electrostatic interaction strength between PEI and the phosphate backbone of double-stranded DNA (dsDNA) is about 24-25 pN by atomic force microscopy 29. This high-strength electrostatic adsorption allows PEI-modified iron oxide NPs to be successfully used for the purification of plasmid DNA in bacterial cells 30 and gene delivery 31. Khamlamoon *et al.* coated PEI with methacrylic acid before PEI modification of iron oxide NPs to increase the positive charge surface on the NPs and then used to extract nucleic acid from the sample 32. Zhen *et al.* modified PEI-superparamagnetic iron oxide (SPIO) with galactose (Gal). Gal can target c-Met siRNA to the tumor, and SPIO modified by PEI and Gal can protect siRNA from degradation 33. In addition, PEI can also be used to modify iron phosphate 34, amino-containing magnetic colloids and thus nucleic acid adsorption through electrostatic interaction 35.

In addition to the two most widely used modifiers above, a variety of other types of modifiers have been developed. For example, cationic lipid was modified on the surface of AuNPs by emulsification/solvent evaporation method. The modified AuNPs can be used for siRNA delivery 36. Arginine-modified AuNPs can also interact with siRNA via electrostatic interactions, leading to efficient siRNA delivery **(Figure 2B)** 37. In addition, cellulose 38, diethylaminoethyl 39, 3-[2-(2-aminoethylamino)-ethylamino]-propyltrimethoxysilane 40, cationic polystyrene 41, and perylene diimide 42 are all good modifiers that adsorb nucleic acids by electrostatic interaction under appropriate salt concentration and pH conditions. It is worth mentioning that Silva *et al.* prepared a new type of hybrid magnetic composite material by wrapping magnetic iron oxide NPs with a special conductive polymer PEDOT chain. In an acidic environment, the nanocomposite can rapidly separate DNA molecules from complex biological samples by electrostatic interaction **(Figure 2C)** 43.

Besides NMs bind to the phosphate backbone of nucleic acids, studies that bind to the bases of nucleic acids have also been reported. Li and Rothberg found that short ss-DNA sequence and high temperature can promote the structural fluctuation of ssDNA, making it easier to unfold and expose bases. This allows negatively charged citrate-coated AuNPs to have a strong ability to electrostatically adsorb ssDNA **(Figure 2D)** 44.

In general, due to the weak electrostatic force formed between NMs and nucleic acids, the number of charges on the surface of NMs can be increased by using surface modifiers, thereby increasing the electrostatic force between NMs and nucleic acids. At present, nucleic acid analysis is widely used by using the electrostatic force formed between nucleic acids and NMs. For example, the use of silica-coated magnetic nanoparticles (MNPs) 21-27 for nucleic acid extraction and the introduction of MNPs into microfluidic chips and automated systems for nucleic acid analysis make the nucleic acid analysis process simpler, and the analysis efficiency and accuracy are also improved. More application scenarios will be shown in Section 5.

## 3. Covalent Interaction between Nanomaterials and Nucleic Acids

In nucleic acid analysis, the covalent coupling between nucleic acids and NMs also occupies a pivotal position. This technology is widely used in many research fields 45. Based on the interaction mechanism described in the currently reported literature, the covalent binding between nucleic acids and NMs is divided into the following six aspects: The first is the direct covalent binding method, which directly establishes a strong covalent bond between nucleic acid molecules and NMs through chemical reactions, thereby achieving a stable connection between them. The second is the biotin-avidin system, which is a highly specific interaction mechanism. Biotin can be pre-modified on nucleic acid molecules, while avidin is usually combined with NMs. The strong interaction between them makes nucleic acid efficiently bind to the surface of NMs. The third is the disulfide bond, as a reversible covalent bond, also plays a significant part in the binding of nucleic acids to NMs. Through specific chemical reactions, disulfide bonds can be introduced between nucleic acids and NMs to achieve the coupling of them. At the same time, the reversibility of this bond also facilitates subsequent operations. The fourth is coordination interaction, which is also an important binding mode. Some NMs have abundant coordination sites on the surface, which can coordinate with specific groups (such as phosphate groups) in nucleic acid molecules to form stable complexes. The fifth is the Azide-alkyne click reaction. Azide and alkyne groups form 1,2,3-triazole bonds. The formed triazole bonds are very stable and have good biocompatibility. Finally, EDC/NHS coupling, by modifying the carboxylic acid or the primary amine group on the surface of the two molecules to be connected, to form a covalent binding mode of the amide bond. The amide bond is a strong covalent bond, and the resulting conjugate has a longer life. In summary, the covalent binding between nucleic acids and NMs is diverse, and each method has its unique advantages and applicable scenarios, thus providing abundant technical means for nucleic acid analysis. The following will discuss these six covalent bonding methods separately, and list some of the current applications in the fifth section.

### 3.1. Direct covalent bonding between nanomaterials and nucleic acids

**Figure 3:**
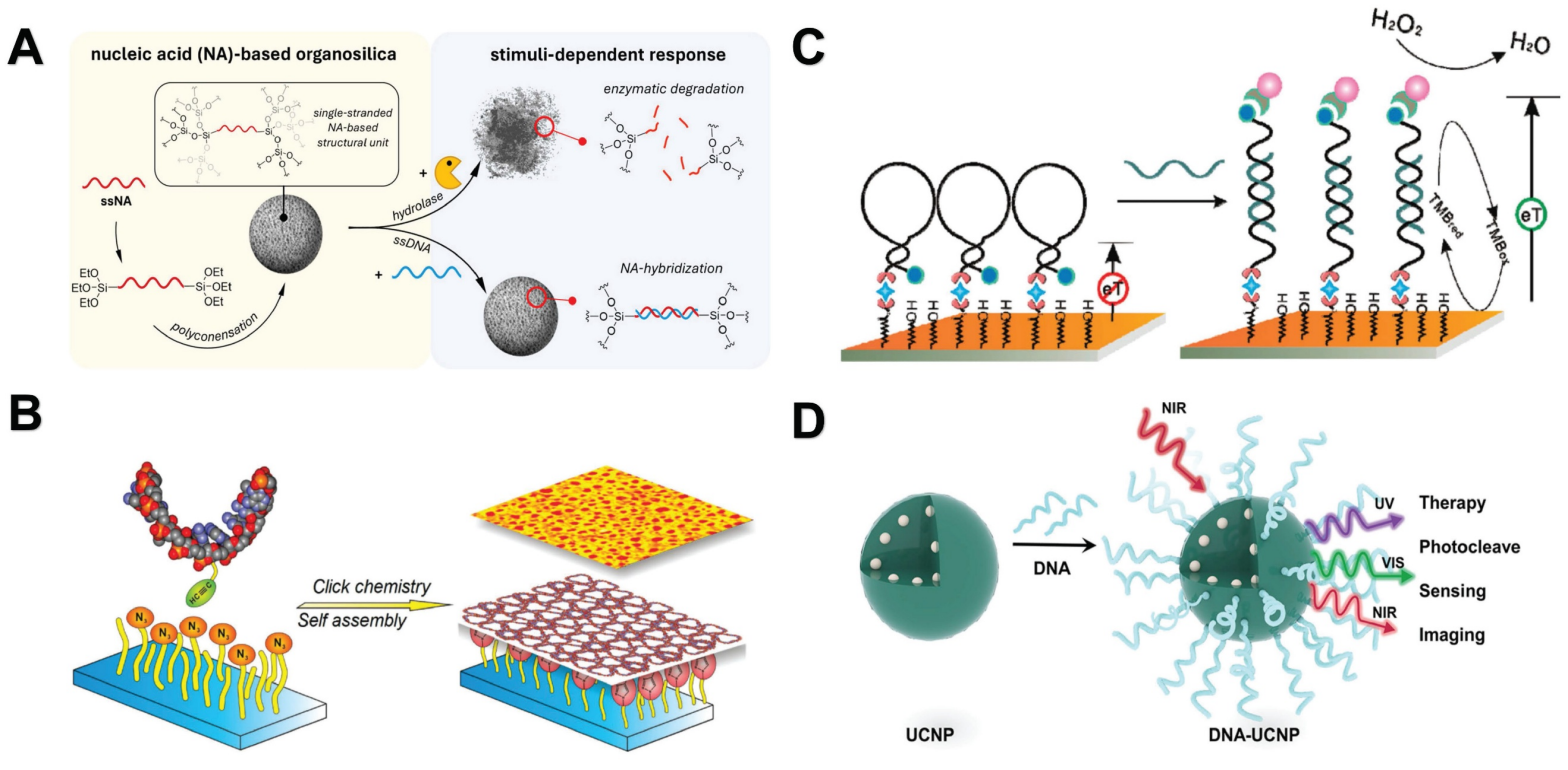
Covalent interaction between NMs and nucleic acids. **A**. ssDNA is covalently embedded in a silica scaffold as an organic bridging group. “Reproduced with permission from American Chemical Society publisher (journal citation 47)”. **B**. The DNA probe was immobilized on the surface of the gold electrode by biotin-avidin connection for nucleic acid detection. “Reproduced with permission from American Chemical Society publisher (journal citation 60)”. **C**. A template-free strategy based on “click” chemistry to fabricate spatially controlled DNA nanopatterns immobilized on surfaces. “Reproduced with permission from American Chemical Society publisher (journal citation 88)”. **D**. UCNPs modified by 3,4-dihydroxyhydrocinnamic acid link DNA via EDC/NHS. “Reproduced with permission from American Chemical Society publisher (journal citation 99)”.

As a basic and powerful chemical bonding method, direct covalent bonding is formed by sharing electron pairs between two or more atoms, thus realizing the delocalization of electrons in a wider range and constructing a stable molecular structure 46. This process not only lays the foundation for the diversity of chemical substances, but also is the core of many scientific phenomena and technology applications. Different from electrostatic interaction, its application range and bonding strength are greater. In nucleic acid analysis, covalent bonds have become one of the preferred way to connect NMs and nucleic acids due to their high stability and control ability. This strong binding force ensures that nucleic acid molecules can be firmly attached to the surface or inside of NMs, which provides a solid foundation for subsequent applications such as biological recognition, sensing, drug delivery, and gene therapy. For example, single-stranded nucleic acids can be covalently embedded into silica, giving silicone NPs a nucleic acid-based sequence-specific chemical, physical and biological responsiveness **(Figure 3A)** 47. In addition, there are AuNPs that can covalently connect siRNA 48 and RNA 49, latex particles that can covalently connect DNA 50, and quantum dot that can covalently bind to RNA 51. Wang *et al.* also synthesized a colloid with a variety of chemical components (polystyrene, polymethyl methacrylate, titanium dioxide, silicon dioxide, and silicon dioxide-methacrylate hybrid materials) that can covalently connect single-stranded oligonucleotides with short sticky ends 52. Through covalent bonds, these NMs that bind to nucleic acids have become extremely effective tools in nucleic acid purification and downstream analysis because of the strong stability of the formed covalent bonds.

### 3.2. Coordination covalent between nanomaterials and nucleic acids

Since the polyphosphoric acid group of DNA can act as a ligand for metal ions to form a common electron pair, the coordination binding between nucleic acid and NMs also belongs to a covalent binding mode. However, there are few related studies, mainly the following, metal organic frameworks UiO-66-NH_2_ modified Fe_3_O_4_ NMs 53, cobalt ferrite NMs 54, and Fe^3+^-iminodiacetic acid-modified silica particles. In the latter, at low pH (≤ 4.0), Fe^3+^ ions can coordinate with DNA phosphate groups to adsorb DNA. At high pH (≥ 5.0), the coordination interaction will be inhibited by hydroxyl groups, and only at high salt concentration can efficient adsorption be obtained 55. Although the coordination interaction is a weak force, it is also a potential way for NMs to extract nucleic acid.

### 3.3. Biotin-avidin mediated covalent between nanomaterials and nucleic acids

The binding between biotin and avidin is also a kind of covalent binding. Researchers have modified both NMs and nucleic acids on their surface to promote the interaction between them. Avidin is a basic biotin-binding glycoprotein from egg white. It consists of four identical subunits, each of which can bind to a biotin molecule. In addition, avidin also has a bacterial analogue streptavidin with a similar structure 56. However, avidin can directly interact with DNA, while streptavidin (a neutral non-glycosylated bacterial analog) does not 57. The following is a review of the current examples of nucleic acid analysis based on NMs using biotin-avidin-specific binding. At present, many researchers combine the nucleic acid probe molecule biotinized with streptavidin-modified magnetic beads to perform downstream analysis of the corresponding target 58-66. For example, Liu *et al.* fixed DNA probes on the surface of avidin-modified electrodes through biotin-avidin bridges, which can detect DNA targets as low as femtomolar and distinguish single mismatches **(Figure 3B)** 60. Horejsh *et al.* used a biotin-streptavidin linker to connect the dual-labeled probe to the microsphere, establishing a fluid array system capable of detecting unlabeled nucleic acids in solution. The system has the specificity of detecting SARS coronavirus 64. In addition, researchers have combined biotinylated peptide nucleic acids with avidin-modified protein NPs to achieve efficient drug delivery 65, 66. The high specificity between biotin and avidin fully ensures the accuracy of the binding between NMs and nucleic acids. Although it is not suitable for nucleic acid extraction in complex samples, it plays an important role in biosensors and gene therapy.

### 3.4. Disulfide bond mediated covalent between nanomaterials and nucleic acids

The disulfide bond is also a kind of covalent bond. The common disulfide bond in nature is formed by the oxidation of two cysteine sulfhydryl groups with the simultaneous release of two electrons. The disulfide bond can be spontaneously formed in vitro 67. In the field of NMs for nucleic acid analysis, researchers have modified NMs and nucleic acids with sulfhydryl groups, thereby promoting the formation of disulfide bonds to enable the interaction between them. For example, thiol-modified siRNA can be combined with thiolated gelatin (tGel) to form poly-siRNA-tGel NMs, for intracellular siRNA delivery to promote the silencing of target genes in cancer cells 68. AuNPs can bind to DNA oligonucleotides through covalent thiol bonds to construct a DNA biosensor for detecting ssDNA 69, 70, with the addition of organic dyes, hybrid materials can also be constructed as molecular beacons 71. In addition, the researchers found that AuNPs-oligonucleotide conjugates prepared using cyclic disulfide bonds showed higher stability than the corresponding conjugates prepared using conventional disulfide bonds 72. For example, the anchoring group used by Letsinger *et al.* binds oligonucleotides to the gold surface (based on 4,5-dihydroxy-1,2-dithiane and epiandrosterone-derived ketoaldehyde), which is the formation of cyclic disulfide bonds 73. In addition, on this basis, there are also trithiol-terminated oligodeoxyribonucleotides and AuNPs conjugates. The stability of these DNA-AuNPs conjugates is significantly higher than that of analogues prepared from monothiols and cyclic disulfide-terminated oligodeoxyribonucleotides 74. Besides these, glassy carbon 75, meso-2,3-dimercaptosuccinic acid modified magnetic NMs 76, paramagnetic Biomag magnetic beads 77 and silica NMs 78 can generate disulfide bonds with thiol-modified nucleic acids because they contain or are modified by thiols. Similar to the biotin-avidin interaction force, the indirect force between NMs and nucleic acids is not suitable for nucleic acid extraction in complex samples. However, due to the stability of disulfide bonds and the ease of thiol modification, disulfide bond can be used as a binding mode between nucleic acid and various NMs, and it is also a common force for nucleic acid modification at the interface.

### 3.5 Azide-alkyne click reaction mediated covalent between nanomaterials and nucleic acids

The azide-alkyne click reaction consists of 1,2,3-triazole bonds formed by the condensation of organic azides and alkyne groups. Because the azide and alkyne functional groups are not chemically reactive to most functions of biomolecules, they have high biocompatibility 79. Azide-alkyne click reaction has been successfully applied to the functionalization of various sugars and proteins 80, 81. In nucleic acid analysis, copper-catalyzed azide-alkyne cycloaddition (CuAAC) and strain-promoted azide-alkyne cycloaddition (SPAAC) are commonly used 79, 82. CuAAC and SPAAC reactions have been widely used in DNA modification 83. For example, acetylene-containing ssDNA can be combined with azide-functionalized AuNPs 84, azide-functionalized superparamagnetic iron oxide NPs 85 and azide-functionalized butyl acrylate NPs 86 to form an ssDNA monolayer for nucleic acid detection. Alkyne-modified dsDNA can be combined with azide-modified glutathione derivative-functionalized AuNPs 87, azide-functionalized Si **(Figure 3C)** 88 and SiO_2_ NPs 89 to prepare chain-like DNA-NPs conjugates, which can capture or deliver DNA for DNA sequencing or nanomedicine applications. Liu *et al.* also reported a dopamine acrylamide ligand for the modification of Lanthanide ion-doped upconversion NPs (UCNPs). The alkenyl group of dopamine acrylamide can be coupled to thiol-modified DNA by thiol-ene click reaction. This thiol-ene coupling reaction is fast, efficient, stable 90. In addition, Siegel *et al.* reported a method of coupling Si and SiO_2_ NMs with DNA by freezing-assisted SPAAC. This simple functionalization method has been shown to greatly improve the overall DNA deposition efficiency. Freezing shortens the reaction time from about 2 days to only a few hours compared to traditional SPAAC, and the DNA density generated on the NP surface is 10 times higher 91. Because of the higher packing density of small molecules on the surface of NPs, the azide-alkyne click reaction is superior to avidin-biotin binding. In addition, as a covalent bond between NPs and connecting molecules, the triazole ring is more stable. However, the azide-alkyne click reaction relies on special chemical modifications and cannot react with natural nucleic acids in complex matrices. It is currently more applied to in vivo labeling and fluorescence imaging of organisms.

### 3.6 EDC/NHS mediated covalent between nanomaterials and nucleic acids

Carbodiimide chemistry is the most commonly used method for covalent modification of free carboxylic acids with primary amines to achieve the purpose of labeling and surface functionalization. The most common carbodiimide is the water-soluble N-(3-dimethylaminopropyl)-N′-ethylcarbodiimide (EDC). EDC is usually used together with N-hydroxysuccinimide (NHS) to accelerate the reaction rate and the final coupling efficiency. DNA or RNA molecules can be chemically synthesized with free carboxylic acid or primary amine groups at the desired nucleotide sites, thereby chemically coupling to the surface of the functionalized material via EDC-NHS 92-94. For example, PNA-bound nanodiamonds 95, DNA-coupled carbon NPs 96, and single-walled carbon nanotubes 97. In addition, there are some composite NMs, such as DNA-bound silica-encapsulated Au nanorods 98 and UCNPs modified by 3,4-dihydroxyhydrocinnamic acid **(Figure 3D)** 99. In addition, researchers have also developed a method to improve the efficiency of conventional EDC reaction by using imidazole to form covalently coupled phosphate amidated ssDNA. The percentage yield of phosphoamide ssDNA was increased by more than 10% compared to conventional methods 100. Because the protein contains natural amino and carboxyl groups, there are more protein analysis based on amide bonds, but less application in the analysis of natural nucleic acids, which can be used as an important way for interface modification or carrier construction of nucleic acids and NMs.

## 4. Other Interaction between Nanomaterials and Nucleic Acids

Apart from the above two most widely used interaction methods, several interaction methods between nucleic acids and NMs have also been used, including hydrophobic interaction, π-π stacking, hydrogen bonding and ion exchange. Hydrophobic interactions widely exist between nonpolar molecules. Although they are not the main driving force for the binding between NMs and nucleic acids, they will have an important impact on the binding. π-π stacking is a non-covalent interaction widely existing between aromatic rings. Nucleic acids have a π-rich structure, making NMs with aromatic ring structure can bind to nucleic acids. Hydrogen bond is a non-covalent interaction between molecules containing hydrogen atoms and atoms with strong electronegativity, which is easy to form and break between nucleic acids and NMs. Ion exchange is a relatively rare interaction method, but due to its effectiveness and operability under specific conditions, it has become a promising way for NMs to adsorb nucleic acids. The following will discuss these four interaction modes respectively, and in the fifth chapter, some examples of the application of NMs in nucleic acid analysis are listed.

### 4.1. Hydrophobic interaction between nanomaterials and nucleic acids

**Figure 4:**
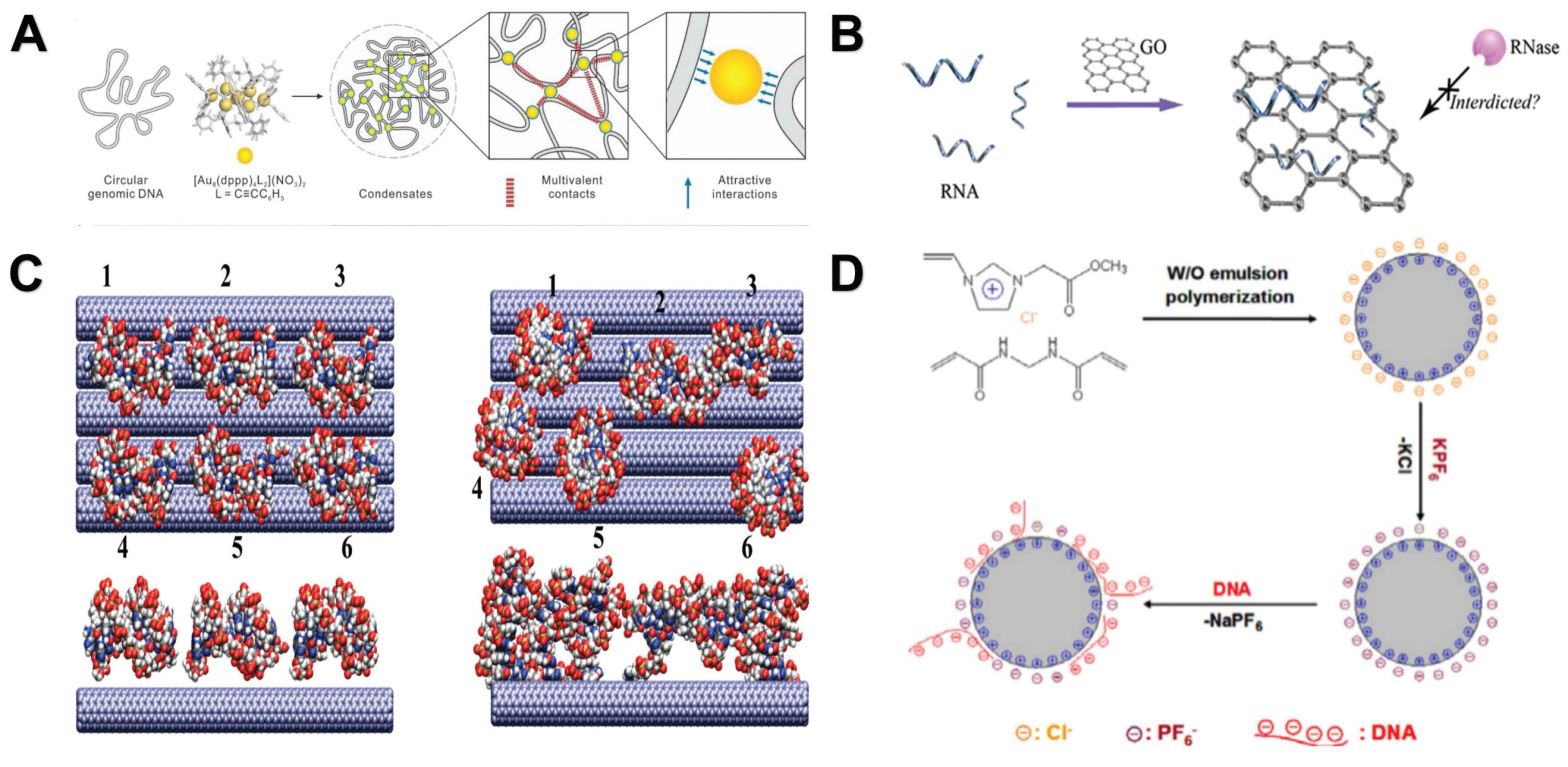
Other ways of interaction between NPs and nucleic acids. **A**. Hydrophobic interactions between the polyvalent AuNCs and bases lead to the condensation of single-stranded nucleic acids into compact structures or condensates. “Reproduced with permission from American Chemical Society publisher (journal citation 102)”. **B**. Adsorption of total RNA by GO. “Reproduced with permission from American Chemical Society publisher (journal citation 106)”.** C**. The assembly of dsDNA fragments on the surface of single-walled carbon nanotubes. The left side represents a snapshot at 0 ns, and the right side represents a snapshot at 28 ns. “Reproduced with permission from American Chemical Society publisher (journal citation 113)”. **D**. A PIL microsphere was synthesized through water-in-oil emulsion polymerization, and the primary force driving the binding of DNA to these PIL microspheres was the ion exchange that occurred between the anionic component of PIL and the DNA. “Reproduced with permission from Elsevier publisher (journal citation 121)”.

The hydrophobic interaction, also referred to as the hydrophobic effect, represents a characteristic of nonpolar molecules (or the hydrophobic segments of amphiphilic molecules). This interaction can propel these molecules to arrange themselves into water-free regions within an aqueous solution. Although hydrophobic interaction is not the main binding force between nucleic acids and NMs, it has an important influence on the interaction between them 101. For example, Li *et al.* found that the dynamic hydrophobic interaction between hydrophobic gold nanoclusters (AuNCs) and DNA bases can make AuNCs move collectively in the limited nanospace of DNA **(Figure 4A)** 102. Wu *et al.* found that the binding between DNA and Graphene oxide (GO) was strongly affected by hydrophobic interactions, and shorter DNA had faster binding efficiency 103. Yeh *et al.* found that when RNA molecules are transported by electrophoresis through a 1.5 nm wide carbon nanotube membrane pore, RNA will adhere to the pore through hydrophobic interaction 104. In summary, the hydrophobic interaction between NMs (AuNCs, GO and carbon nanotube membrane pore) and nucleic acids can be used to apply nucleic acid downstream analysis.

### 4.2. π-π stacking interaction between nanomaterials and nucleic acid

π-π stacking represents a distinctive spatial organization of aromatic or aromatic-related compounds, characterized by a weak attractive force acting between their aromatic ring systems. Nucleotide molecules are composed of nitrogen-containing bases containing pentose and phosphoric acid. It has a π-rich electronic structure similar to aromatic rings. Hence, it facilitates forming π-π stacking interactions between nucleic acids and other aromatic ring-structured molecules and materials 105. GO is a material that can immobilize nucleic acids through π-π stacking. By combining GO with nano-magnetic beads, nucleic acids can be rapidly purified and protected from complex samples **(Figure 4B)** 106. The adsorption of dsDNA on GO is due to the partial deformation of the DNA double helix on the surface of GO, and the π-π stacking between the DNA terminal base pairs and the carbon rings. At the same time, the binding affinity of dsDNA to GO was enhanced by the hydrogen bonds formed between GO and the oxygen-containing groups of DNA bases 107. Xu *et al.* found that the ssDNA fragment captures the oxidized groups on the surface of GO through hydrogen bonding interactions, and then relaxes the configuration to maximize the π-π stacking between the aromatic rings of the nucleobase and the aromatic rings on the surface of GO 108. The content of hydrogen bonds will be introduced in detail in the next section. By using this characteristic of GO, Ahour *et al.* used GO as an electrode modifier to construct an electrochemical biosensor, and the probe ssDNA was adsorbed on the surface of GO/pencil graphite electrode by π-π interaction for target detection 109. Duan *et al.* developed a surface enhanced Raman scattering based platform for anchoring DNA to GO-Au@Ag NMs through π-π interaction. Through partial hybridization of magnetic probes, target DNA can be separated and concentrated from samples 110. In addition, researchers have found that carbon nanotubes 111 and carbon nanospheres 112 can also interact with DNA by π-π stacking interaction **(Figure 4C)** 113 for nucleic acid purification and analysis.

### 4.3. Hydrogen bonds interaction between nanomaterials and nucleic acid

The hydrogen bond represents the attractive force between a proton donor, X-H and a proton acceptor, Y. Although hydrogen bonds are weaker than chemical bonds, this relative weakness underscores their significance. Due to their ease of formation and disruption at room temperature, hydrogen bonds are crucial in the chemistry of life, playing a pivotal role in various biological processes 114. In the application of the interaction between NMs and nucleic acids, hydrogen bonds exist in the contact surface of various materials and nucleic acids because they are extremely easy to produce. Moreover, due to the easy breaking of hydrogen bonds, it is also easy to elute the nucleic acid from the surface of the material. Previously, it has been mentioned that GO can bind to nucleic acids in a π-π stacking manner, but it has also been found that hydrogen bonding is also the main force for nucleic acids to bind to the surface of GO 115 and nucleic acids can be eluted from the surface of the material by using urea. Some researchers have found that hydrogen bonding is one of the main driving forces for the binding of nucleic acids to silica surfaces 116. For example, in the silicon fluid microchip for extracting and concentrating DNA introduced by Christel *et al.*, it was found that the formation of hydrogen bonds between silica and DNA in solution was the main driving force for DNA to adsorb to silica 117. In the capillary filled with silica resin of purified DNA used by Tian *et al.*, it was also found that the formation of hydrogen bonds between silica and DNA in solution was the main driving force for DNA adsorption to silica 118. Additionally, Melzak *et al.*, conducted research on the adsorption of silica by plasmid and chromosome dsDNA, and the results indicated that the establishment of intermolecular hydrogen bonds within the DNA-silica contact layer was a major factor contributing to the overall adsorptive force 119. The rapid formation of hydrogen bonds and the nature of easy breakage ensure that GO and silica can quickly extract nucleic acid samples from complex matrices, and can elute the nucleic acids adsorbed on the surface after applying appropriate chemical means, making the analysis of nucleic acids more convenient.

### 4.4. Ion exchange interaction between nanomaterials and nucleic acid

An ion exchange reaction is defined as the process in which cations or anions in the functional groups of an ion exchanger undergo a reversible exchang with similar ions present in a solution 120. Nucleic acids can be combined with NMs through this reaction. For example, Wang *et al.* prepared a new type of polymer ionic liquid (PIL) microsphere by water-in-oil emulsion polymerization. The rapid ion exchange between PIL and the anionic part of the DNA fragment reached an exchange equilibrium within 1 minute, which can adsorb DNA at low salt concentrations and recover DNA at high salt concentrations. It has been successfully used to adsorb plasmid DNA from *E. coli* cell culture because it is operable and effective under harsh conditions. Ion exchange-based DNA adsorption is superior to electrostatic interaction-based adsorption **(Figure 4D)** 121. Therefore, it is speculated that this force may replace the electrostatic interaction in the future and become the main research direction in the field of nucleic acid analysis assisted by NMs.

## 5. Application of Nanomaterials in Nucleic Acid Analysis

The role of NMs in nucleic acid analysis is mainly reflected in the adsorption of nucleic acids through the various forces mentioned above, so as to achieve the purpose of separating and purifying nucleic acids from complex samples. At the same time, magnetic NMs have become the most widely used NMs because of their large specific surface area, excellent magnetic properties and the ability to be controlled by external magnetic fields. The following introduces some practical applications of NMs in the field of nucleic acid analysis. At present, the more developed applications are mainly reflected in three aspects, including magnetic bead nucleic acid extraction kits, microfluidic chips and some automated nucleic acid analysis systems.

### 5.1. Nanomaterials-assisted nucleic acid extraction

Due to the advantages of easy operation and good dispersibility of magnetic nanomaterials (MNMs), many magnetic bead kits are widely used in the market. Nucleic acid extraction based on magnetic beads is less time-consuming and more convenient than traditional centrifugal adsorption column extraction. For example, Ravlo *et al.* developed a detection kit based on NAxtra magnetic beads. NAxtra magnetic beads are paramagnetic iron oxide NPs coated with silica, which can provide high-quality nucleic acids within 14 minutes 122. Li *et al.* developed a magnetic bead nucleic acid extraction kit based on micron-sized MBs@SiO_2_ particles with a lysis time of 5 minutes 123. Diefenbach *et al.* found that the Qiagen QIAamp minElute cfDNA mini kit represents the best magnetic bead kit by comparing a variety of kits 124. Kleines *et al.* investigated the usability of the Chemagic virus DNA/RNA kit and discovered that it possessed the capability to swiftly and effectively extract viral DNA (such as cytomegalovirus and hepatitis B virus) and viral RNA (like hepatitis G virus) from human samples 125.

**Figure 5:**
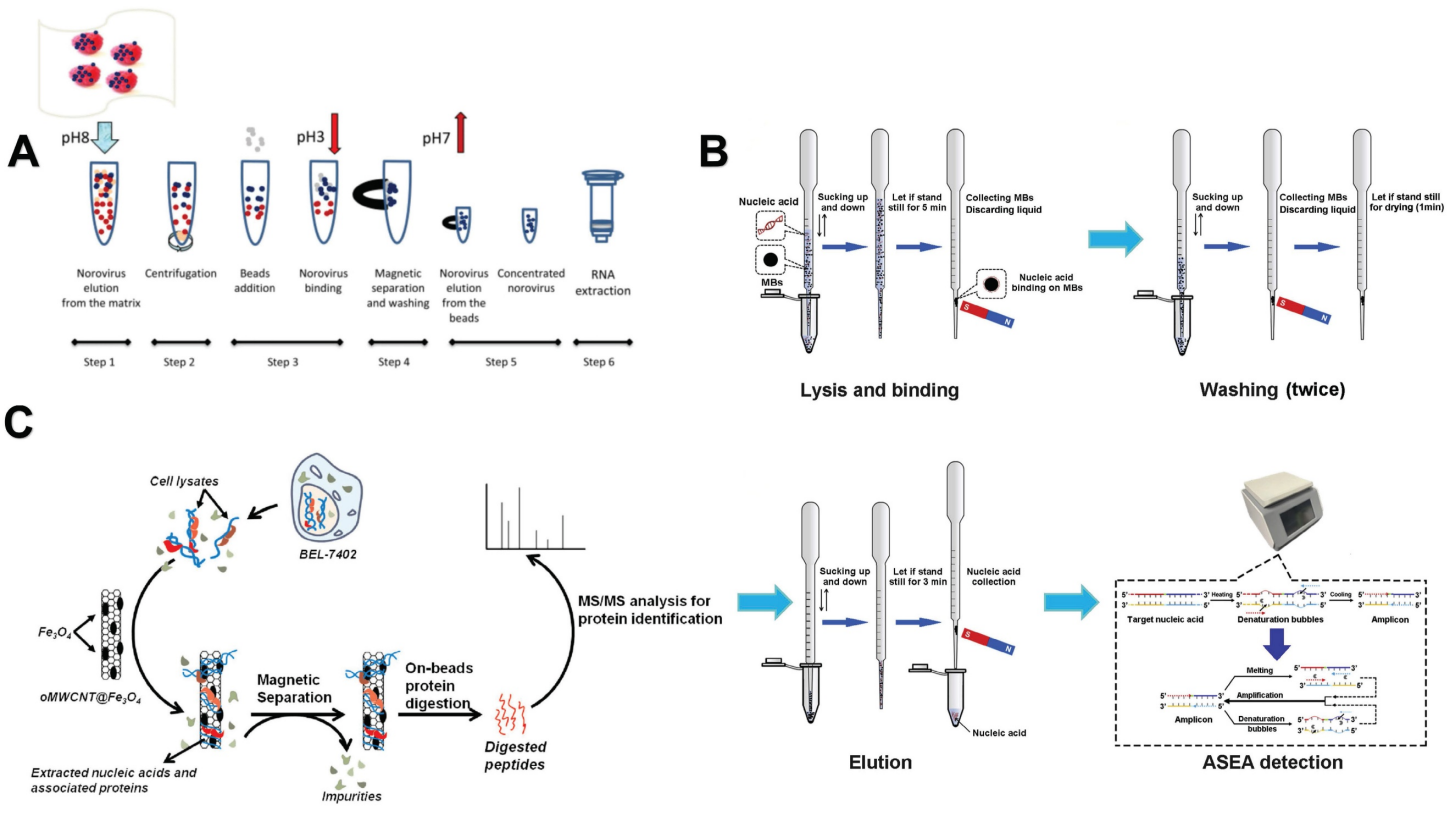
Nanomaterials-assisted nucleic acid extraction.** A**. The method for extracting and concentrating norovirus involves utilizing magnetic silica beads. Prior to RNA extraction, these beads are used to purify the virus (represented by blue dots) from contaminants present in the matrix and soil (represented by brown and red dots), with the magnetic silica beads (depicted as gray dots) facilitating this separation process. “Reproduced with permission from Springer Nature publisher (journal citation 126)”. **B**. Nucleic acid extraction and accelerated denaturation bubbles mediated strand exchange amplification (ASEA) detection within the Pasteur pipette system. The schematic diagram includes the cleavage and binding stages of nucleic acid extraction in the Pasteur pipette system, the washing stage involved in the extraction process, the elution step of nucleic acid recovery from the Pasteur pipette system, and the ASEA reaction. “Reproduced with permission from Elsevier publisher (journal citation 134)”. **C**. Steps for the extraction of nucleic acid from cells using oMWCNT@Fe_3_O_4_. “Reproduced with permission from American Chemical Society publisher (journal citation 137)”.

In addition to commercially available kits, many similar nucleic acid extraction methods based on NMs have been developed. For example, Raymond *et al.* developed a new method using fine magnetic silica beads to extract human norovirus RNA from frozen raspberries. The effect is similar to that of commercially available kits **(Figure 5A)** 126. Fukuchi *et al.* employed silica-coated magnetic beads, which incorporated Fe-based amorphous alloys, as an alternative to conventional iron oxide NPs for the extraction of genomic DNA or RNA, resulting in increased yields 127. Fan *et al.* utilized an innovative three-step grafting technique to coat the surface of MNPs with silica. These specially prepared particles were effectively utilized for the extraction of plasmid DNA, achieving an elution rate superior to that of commercially available magnetic beads 128. Bulgakova *et al.* synthesized a nanocomposite composed of MNPs and nylon 6 (MNP@Ny6). The oligonucleotide was immobilized on MNPs@Ny6 by Ultraviolet induction for magnetic separation of nucleic acids, which was successfully applied to the analysis of yeast RNA with high specificity 129. Wang *et al.* employed MNPs for the simultaneous extraction of DNA and RNA from bacteria. They formulated a lysis buffer to facilitate the release of nucleic acids and their adsorption onto the MNPs. To eliminate contamination from proteins and carbohydrates, two washing buffers were utilized. Finally, the nucleic acids were eluted using water that was free of DNase and RNase 130. Amino-functionalized MNPs can capture nucleic acid from various samples by hybridization with complementary target sequences. Galluzzi *et al.* tested a DNA extraction method based on silica-coated superparamagnetic NPs, and successfully extracted DNA from cultured *Alexandrium catenella* cells to harmful algal bloom species 131. Zhao *et al.* synthesized MNPs coated with polyaminoester and carboxyl groups, which they utilized for RNA extraction. This innovative method integrates the cleavage and binding steps into a single process, enabling the purification of viral RNA from multiple samples in just 20 minutes 132. Sun *et al.* developed a magnetic iron oxide nanoparticle functionalized with 4-carboxyphenylboronic acid (CPBA-MNPs). CPBA-MNPs can be used to extract a variety of genomic DNA and RNA with high quality and the adsorption capacity of RNA can be enhanced in the presence of divalent cations 133.

Interestingly, in addition to the above-mentioned traditional NMs-based nucleic acid extraction methods, some new methods have been developed in recent years. For example, Kang *et al.* established a new Pasteur pipette system to extract pathogen nucleic acid from a variety of samples through MNP. It takes about 15 minutes and does not involve toxic organic reagents or electrical equipment, making it fast and convenient **(Figure 5B)** 134. Zhou *et al.* established a one-stop genomic DNA (gDNA) extraction technology based on salicylic acid (SA) MNPs. SA is a chemical ligand with carboxyl and phenolic functional sites and has excellent adsorption properties 135. Shan *et al.* developed an RNase-free method for the purification of plasmid DNA (pDNA) directly from RNA-containing *E. coli* crude lysates using carboxyl-functionalized MNPs. This method utilizes the difference in adsorption of pDNA and RNA on the surface of MNPs at different temperatures 136. Zhang *et al.* used magnetic oxidized multi-walled carbon nanotubes (oMWCNT@Fe_3_O_4_) to efficiently extract cellular nucleic acid. After adding oMWCNT@Fe_3_O_4_ into the cell lysate, the intracellular nucleic acid was wrapped around the carbon nanotubes, and the nucleic acid-related protein (NAaP) in the cell was also attracted accordingly. With the help of the magnet, nucleic acid and NAaP were isolated from the cell lysate **(Figure 5C)** 137.

### 5.2. Nanomaterials-assisted microfluidic nucleic acid analysis

**Figure 6:**
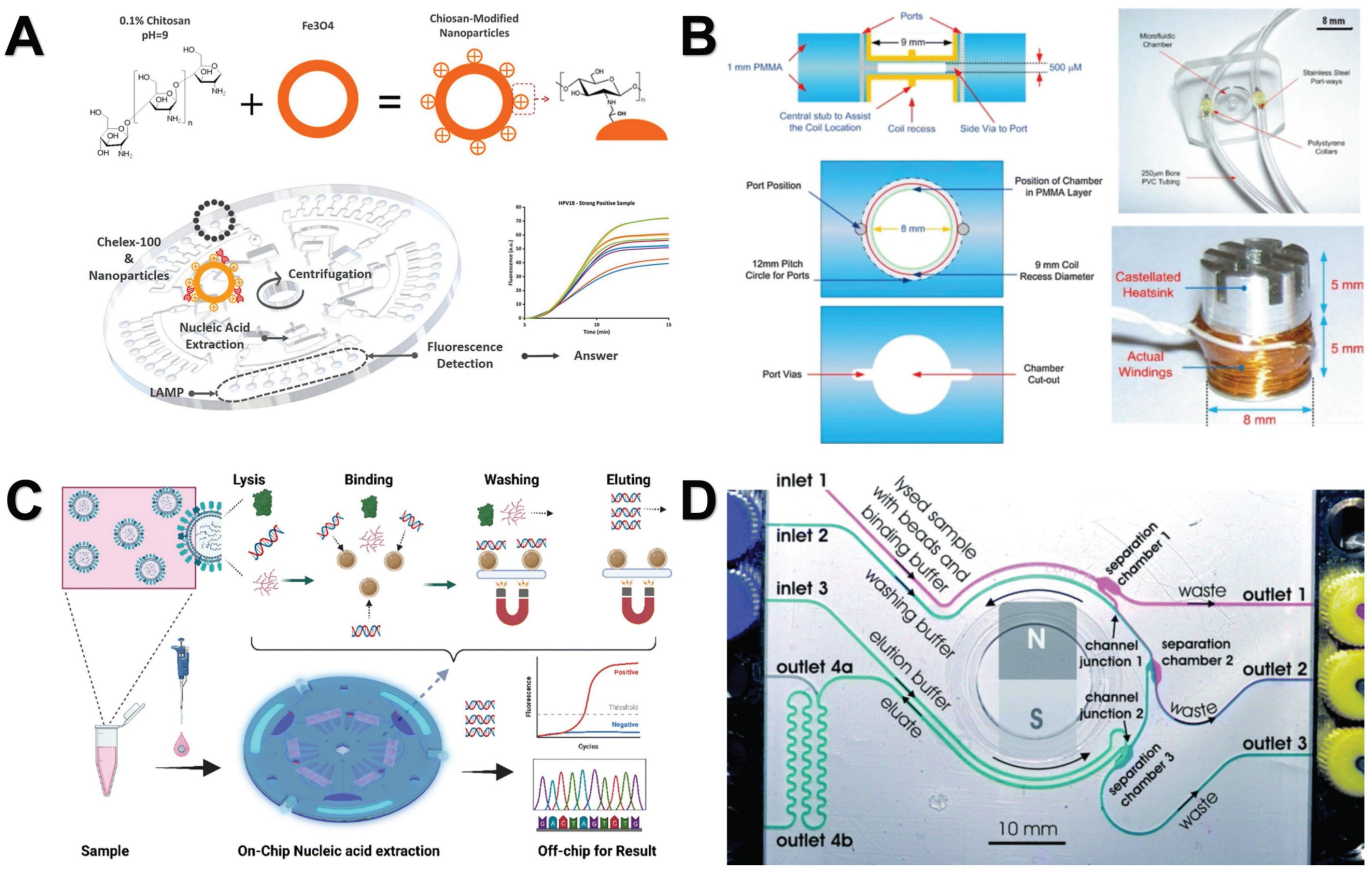
Nanomaterials-assisted microfluidic nucleic acid analysis. **A.** microfluidic chip integrated with chitosan-modified magnetic microspheres, and expanded isothermal amplification and real-time fluorescence detection. “Reproduced with permission from Elsevier publisher (journal citation 140)”.** B**. Detailed construction of a magnetic bead-based microfluidic chip device for extracting DNA from bacteria. “Reproduced with permission from Springer Nature publisher (journal citation 141)”. **C**. A microfluidic system capable of active centrifugation, magnetic beads are pre-embedded in the chip. “Reproduced with permission from ROYAL SOCIETY OF CHEMISTRY publisher (journal citation 142)”. **D.** Schematic illustration of continuous microfluidic DNA extraction using phase transfer magneto swimming. “Reproduced with permission from ROYAL SOCIETY OF CHEMISTRY publisher (journal citation 143)”.

The development of microfluidic devices is devoted to the direct integration of nucleic acid extraction steps from the original sample into the chip. This innovation has greatly improved the portability and speed of the operation and is currently being widely explored for the analysis of nucleic acid samples 138. By combining NMs with microfluidic chips, the nucleic acid analysis process can be further accelerated and simplified, making it faster and more convenient 139. For example, Zhao *et al.* employed chitosan-modified magnetic microspheres for pH-induced nucleic acid extraction and integrated this technique into a centrifugal microfluidic chip. Additionally, they incorporated isothermal amplification and real-time fluorescence detection into the system. The entire operational process is managed through the use of centrifugal force and magnetic control **(Figure 6A)** 140. Azimi *et al.* introduced a chip device based on magnetic beads, using a magnetic micromixer, which can effectively mix whole blood samples with lysis buffer and binding buffer in a circular microchamber, and then efficiently extract nucleic acids **(Figure 6B)** 141. Li *et al.* introduced a novel active centrifugal microfluidic system in which the necessary reagents and magnetic beads are pre-installed inside the chip. Upon introducing the sample, the entire process of nucleic acid purification can be accomplished in just 30 minutes **(Figure 6C)** 142. Karle *et al.* introduced a novel platform for continuous DNA extraction and purification based on superparamagnetic beads by phase-transfer magnetic electrophoresis, achieving continuous biomolecular processing **(Figure 6D)** 143.

In summary, the microfluidic chip based on MNMs has broad application prospects in the field of nucleic acid analysis. In the field of nucleic acid purification, it has been developed to extract a variety of nucleic acids 144, including gDNA 145, ssDNA 146, dsDNA 147 and various free DNA 148. At the same time, the effect of nucleic acid purification can be further improved by applying centrifugal force 149, voltage 150, magnetic field 151 and other means. In terms of nucleic acid detection, microfluidic chips based on MNMs can achieve qualitative 152 and quantitative detection 153. In addition, compared with the traditional microfluidic chips based on hard magnets, soft magnet microfluidic chips that can be plasticized have also been developed 154.

### 5.3. Nanomaterials-assisted automated nucleic acid analysis

**Figure 7:**
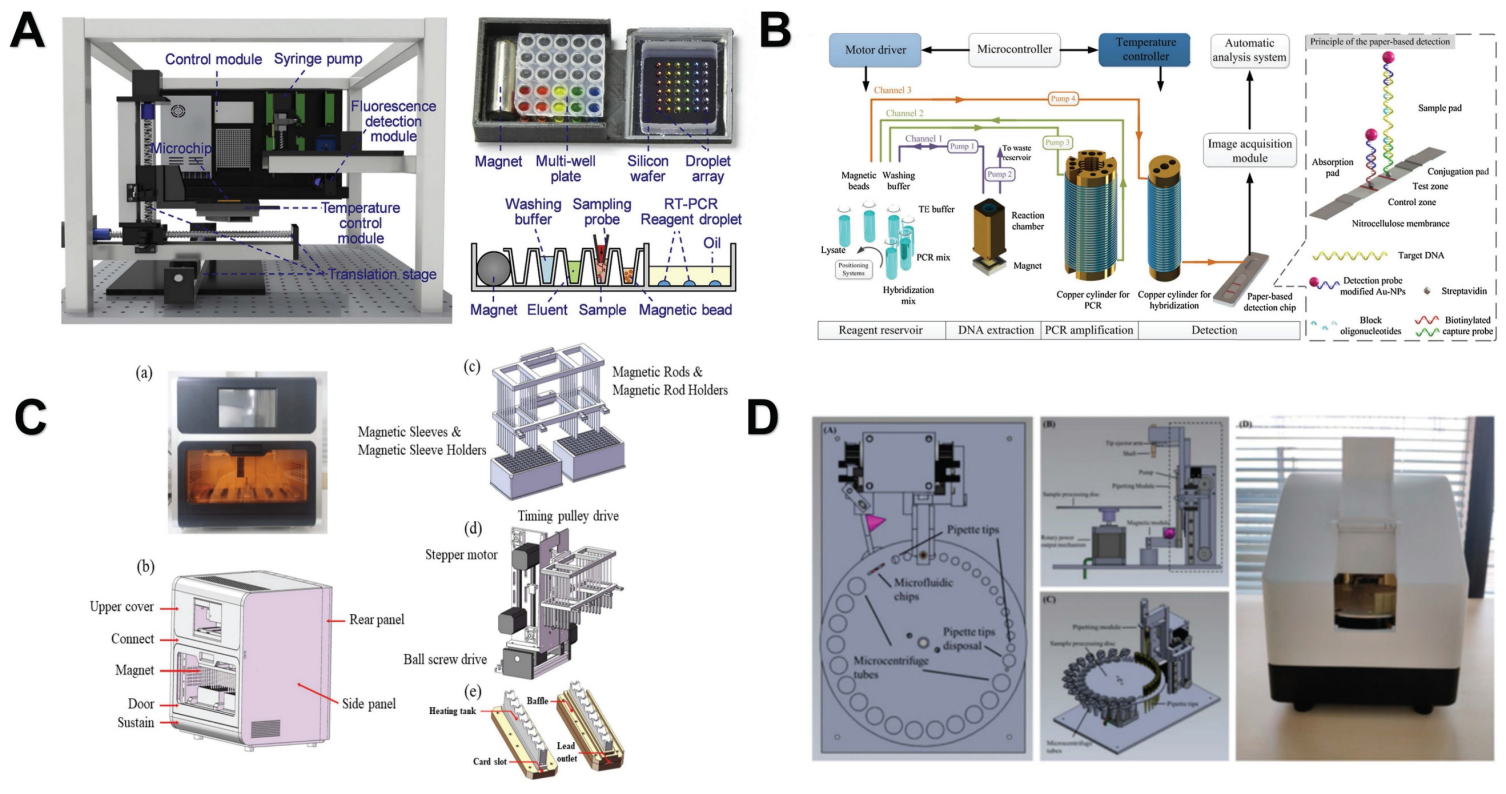
Nanomaterials-assisted automated nucleic acid analysis. **A**. A schematic representation of an automated, fully integrated nucleic acid analyzer that leverages microfluidic liquid handling robotics technology. “Reproduced with permission from Elsevier publisher (journal citation 157)”. **B**. Schematic of an automated pathogen identification platform. “Reproduced with permission from Elsevier publisher (journal citation 158)”. **C**. (a) Frontal perspective of the nucleic acid extraction device. (b) Schematic layout of the external design of the nucleic acid extraction instrument. (c) Separation module utilizing magnetic rods. (d) Mechanical actuation module. (e) Structure of the heating trough. “Reproduced with permission from MDPI publisher (journal citation 159)”. **D**. Schematic diagram of the automated miniaturized rotary sample preparation device. (a-c) Top, front, side and isometric view of the device. (d) Actual image of the device with all modules integrated in a portable box. “Reproduced with permission from MDPI publisher (journal citation 161)”.

The researchers found that the efficiency of magnetic bead-based microfluidic chips in DNA extraction is comparable to that of traditional manual methods, and its advantages are mainly reflected in simplifying complex operation procedures and shortening extraction time 144. Although the microfluidic chip has simplified some of the nucleic acid analysis process, it still contains more manual steps. To further improve the convenience and efficiency of nucleic acid analysis, researchers have developed an automated nucleic acid analysis system based on microfluidic chip technology. At present, these automated extraction systems are gradually replacing traditional nucleic acid extraction methods because they are simple to operate and greatly reduce manual operation time 155. In addition, a significant advantage of using magnetic particles for nucleic acid separation is that it can support automated processes 156. Therefore, the combination of NMs and automation systems makes the nucleic acid extraction process more efficient and convenient. For example, Lin *et al.* created an automated and fully integrated nucleic acid analyzer by integrating automated liquid handling robot technology with microfluidic droplet-based real-time PCR detection. This analyzer is capable of performing multiple tasks, such as sample introduction, nucleic acid extraction utilizing magnetic solid-phase extraction, reverse transcription and droplet generation, PCR amplification, as well as real-time dual fluorescence detection of droplet arrays **(Figure 7A)** 157. Fu *et al.* constructed an integrated genetic analysis platform by using magnetic silica beads to separate DNA from lysates, which can completely automate sample pretreatment, nucleic acid amplification, and endpoint detection, followed by PCR denaturation, annealing and extension stages, using nucleic acid paper detection chip for final detection and automatic analysis of the results by software **(Figure 7B)** 158. Li *et al.* proposed a comprehensive design of a rapid automated nucleic acid extraction system based on magnetic separation. Using automated systems and manual methods for sample extraction, high-purity nucleic acids can be isolated from bacteria, blood, and animal tissues for Reverse Transcription-PCR detection **(Figure 7C)** 159. Politza *et al.* developed a programmable electromagnetic actuator with a highly scalable motion space that can successfully extract viral RNA from 50 μL of plasma samples 160. Tong *et al.* developed an automated miniaturized nucleic acid extraction device based on magnetic bead method, which has the advantages of flexibility, time saving (10 minutes) and simple nucleic acid sample preparation **(Figure 7D)** 161.

In summary, by introducing MNMs into automated systems 161, 162, especially those microfluidic chips based on NMs, they can be accurately driven and regulated by electromechanical control systems 163. At the same time, a variety of DNA and RNA can be efficiently separated from a variety of biological samples 164-167 and complete and integrated nucleic acid analysis can be achieved after integrating nucleic acid amplification and detection devices 168. This not only greatly simplifies the traditional nucleic acid analysis process, but also significantly improves the analysis efficiency and accuracy, and has broad development prospects.

## 6. Conclusion and Foresights

For many years, NMs have garnered extensive use and research attention owing to their distinctive characteristics, including minute size, surface adaptability, enhanced solubility and multifaceted utility. A range of NMs, including encompassing MNMs, metal NMs, silica NMs, polymeric NMs, carbon nanotubes and quantum dots, have been widely applied in the extraction and detection of nucleic acids, potentially paving the way for novel and promising application areas 2, 169, 170. MNMs possess a non-porous nature similar to latex particles, ensuring that reagents interact exclusively with the surface of the support. Additionally, they provide a substantial surface area conducive to DNA attachment. Leveraging a magnet allows for the swift and effortless separation of magnetic beads from the solution. During the removal or exchange of the solution, the beads remain anchored in a tube with the aid of a magnet, thereby eliminating the need for centrifugation during DNA coupling and hybridization reactions, resulting in significant time savings 171-173.

The wide application of NMs in the field of nucleic acid extraction technology has greatly promoted the progress and development of the nucleic acid extraction technology. The interaction mechanism between NMs and nucleic acid molecules are complex and diverse, mainly including electrostatic interaction, various forms of covalent binding (direct covalent binding, biotin-avidin system, disulfide bond connection, coordination interaction, azide-alkyne click reaction, EDC/NHS coupling), π-π stacking, hydrogen bond formation, hydrophobic interaction, and ion exchange. Among these interactions, electrostatic interaction and covalent binding have become the two most widely used mechanisms due to their high efficiency and stability. The further integration of NMs with advanced systems such as microfluidic chips and biosensors not only improves the sensitivity and speed of nucleic acid detection but also injects new vitality into the development of rapid detection technology. In this paper, we systematically reviewed the main interaction modes between NMs and nucleic acid molecules and demonstrated the diversified applications of NMs in the field of nucleic acid analysis through a series of examples, such as improving extraction efficiency and enhancing detection signals.

In conclusion, this paper aims to provide valuable reference and inspiration for the future development of more rapid, efficient and reliable nucleic acid analysis technology by reviewing the various ways of interaction between NMs and nucleic acid molecules and their application cases in nucleic acid analysis, as well as the latest research results of NMs in the field of nucleic acid analysis, so as to play a more important role in environmental monitoring, drug development, disease diagnosis and food safety.

## Figures and Tables

**Table 1:** The summary of nanomaterials' interaction with nucleic acids.

Main modes	Direct / Indirect	Nanomaterials (NMs) or NMs surface modifier types	Target	Reference
Electrostatic interaction	Direct	Indium tin oxide	Nucleic acid	14
		Au	ssDNA, RNA	15
		Organic clay and montmorillonite	DNA	16
		Spinel ferrite	Nucleic acid	17
		Enamelin	Nucleic acid	18
		Nickel ferrite	Nucleic acid	19
	Indirect	Silica-modified magnetic particles	Nucleic acid	21, 23-27
		Polyethyleneimine modified NMs	dsDNA, siRNA	29-35
		Arginine-modified gold NMs	siRNA	36
		Lipid-modified gold NMs	siRNA	43
		Cellulose-modified NMs	RNA	37
		PEDOT modified iron oxide NMs	DNA	38
		Diethylaminoethyl modified NMs	DNA	39
		3-[2-(2-aminoethylamino)-ethylamino]-propyltrimethoxysilane modified NMs	DNA	40
		Citrate modified AuNPs	ssDNA	41
		Polystyrene modified NMs	Thiophosphate oligonucleotides	42
		Perylene diimide modified NMs	DNA	44
Covalent bond	Direct	Silica	ssDNA, RNA	47
		Au	RNA	48, 49
		Latex particle	ssDNA, RNA	50
		Quantum dot	RNA	51
		Hybrid colloids	ssDNA with short sticky ends	52
Coordination interaction	Direct	Cobalt ferrite	DNA	54
	Indirect	Metal organic frameworks UiO-66-NH_2_ modified Fe_3_O_4_ NMs	Nucleic acid	53
		Fe^3+^-iminodiacetic acid modified silica particles	DNA	55
Biotin-avidin	Indirect	Avidin-modified NMs	Biotin-modified nucleic acid	58-66
Disulfide bond	Indirect	Thiol-modified gelatin	Thiol-modified nucleic acids	68
		Thiol-modified AuNPs		69-74
		Thiol-modified glassy carbon		75
		DMSA modified MNPs		76
		Thiol-modified paramagnetic Biomag magnetic beads		77
		Thiol-modified silica NMs		78
Azide-alkyne click reaction	Indirect	Azide-modified NMs	Alkyne-modified nucleic acid	84-91
EDC/NHS coupling	Indirect	Nanodiamonds	PNA	95,
		Carbon NPs	DNA	96
		Single-walled carbon nanotubes	DNA	97
		Silica-encapsulated Au nanorods	DNA	98
		3,4- dihydroxyhydrocinnamic acid modified Lanthanide ion-doped upconversion NPs	DNA	99
Hydrophobic interaction	Direct	Graphene oxide (GO)	DNA	103
		Carbon nanotube	RNA	104
π-π stacking	Direct	GO	DNA	106-110
		Carbon	Nucleic acid	111-113
Hydrogen bond	Direct	GO	Nucleic acid	115
		Silica	DNA	117-119
Ion exchange	Direct	Polymer ionic liquid microspheres	DNA	121

## Abbreviations

NPs: Nanoparticles
NMs: Nanomaterials
MNPs: Magnetic nanoparticles
MNMs: Magnetic nanomaterials
ITO: Indium tin oxide
PEI: Polyethyleneimine
ssDNA: Single-stranded DNA
dsDNA: Double-stranded DNA
gDNA: Genomic DNA
Gal: Galactose
tGel: Thiolated gelatin
AuNCs: Gold nanoclusters
AuNPs: Gold nanoparticles
GO: Graphene oxide
PIL: Polymer ionic liquid
MNP@Ny6: Magnetic nanoparticles and nylon 6
CPBA-MNPs: Magnetic iron oxide nanoparticle functionalized with 4-carboxyphenylboronic acid
SA: Salicylic acid
pDNA: Plasmid DNA
oMWCNT@Fe_3_O_4_: Magnetic oxidized multi-walled carbon nanotubes
NAaP: Acid-related protein
ASEA: Accelerated denaturation bubbles mediated strand exchange amplification
UCNPs: Lanthanide ion-doped upconversion NPs
CuAAC: Copper-catalyzed azide-alkyne cycloaddition
SPAAC: Strain-promoted azide-alkyne cycloaddition
EDC: N-(3-dimethylaminopropyl)-N′-ethylcarbodiimide
NHS: N-hydroxysuccinimide
